# Improving butenyl-spinosyn production in *Saccharopolyspora pogona* through combination of metabolic engineering and medium optimization

**DOI:** 10.3389/fmicb.2025.1561042

**Published:** 2025-04-23

**Authors:** Xueli Zhao, Haisong Lu, Sen Peng, Haifeng Hang, Waleed Aldahmash, Muath Q. Al-Ghadi, Weihua Tang, Jiang Pei, Wan Xun, Meijin Guo, Ali Mohsin

**Affiliations:** ^1^State Key Laboratory of Bioreactor Engineering, East China University of Science and Technology, Shanghai, China; ^2^Shanghai Baying Bio Technology Co., Ltd., Shanghai, China; ^3^Department of Zoology, College of Science, King Saud University, Riyadh, Saudi Arabia; ^4^Shanghai Morimatsu Pharmaceutical Equipment Engineering Co Ltd., Shanghai, China

**Keywords:** *Saccharopolyspora pogona*, butenyl-spinosyn, genomics-based metabolic engineering, medium optimization, targeted metabolomic analysis

## Abstract

Butenyl-spinosyn is a high-quality biological insecticide produced by *Saccharopolyspora pogona* that effectively targets a broad range of insect pests. However, the large-scale production of this insecticide is hindered by its low yield. Herein, based on prior comparative genomic analysis, five mutations were individually overexpressed in aG6. Subsequently, the combinatorial overexpression of *sp1322* (encoding NAD-glutamate dehydrogenase) and *sp6746* (encoding dTDP-glucose 4,6-dehydratase) in aG6 resulted in strain O1322-6746. The production of butenyl-spinosyn in O1322-6746 was 77.1% higher than that in aG6. Comparative targeted metabolomic analysis uncovered that O1322-6746 exhibited increased metabolic flux toward butenyl-spinosyn precursors. Furthermore, single-factor experiments, Plackett-Burman analysis and response surface methodology were performed to optimize the fermentation medium for O1322-6746. Ultimately, butenyl-spinosyn production was enhanced to 298.5 mg/L in a 5-L bioreactor, marking the highest yield ever reported. This work demonstrated that combining metabolic engineering with medium optimization is an effective strategy to improve butenyl-spinosyn production.

## 1 Introduction

Butenyl-spinosyn, a bioinsecticide produced by *Saccharopolyspora pogona* (*S. pogona*), exhibits environmental friendliness and a broad insecticidal spectrum (Tang et al., 2021; Rang et al., 2022). It is noteworthy that butenyl-spinosyn has a broader insecticidal spectrum and greater insecticidal activity than spinosyn (He et al., 2021; Hu et al., 2022). To boost the industrial implementation of butenyl-spinosyn, recent efforts focused on enhancing its production. To date, various strategies have been employed to improve the yield of butenyl-spinosyn, including random mutagenesis (Zhao et al., 2024), knockout of competing gene clusters (He et al., 2021; Rang et al., 2021), and modulation of transcriptional regulators (He et al., 2020; Tang et al., 2021). However, because of its intricate biosynthesis pathway, the highest reported yield of butenyl-spinosyn was 154.1 ± 10.98 mg/L (Rang et al., 2022), which was relatively low. Therefore, it is imperative to explore further ways to improve the production of butenyl-spinosyn (Li et al., 2018; Hu et al., 2022).

Previously, many strain improvements were generated by random mutagenesis methods (Chen et al., 2020; Zhang et al., 2019). In our previous study, we obtained *S. pogona* aG6 from wild-type *S. pogona* (WT) through random mutagenesis, and aG6 exhibited a 4-fold increase in butenyl-spinosyn production (Zhao et al., 2024). Following this, comparative genome analysis between aG6 and WT indicated that the mutated genes were primarily associated with the metabolism of amino acids, carbohydrates, lipids, and coenzymes (Zhao et al., 2024). Given these results and recent advancements in genetic manipulation technology, metabolic engineering of mutated genes attained from comparative analysis could be a rational strategy for improving butenyl-spinosyn production.

Nowadays, comparative genome analysis has been utilized in discovering gene targets for improved production of desired secondary metabolites. For example, a comparative genome analysis of *Streptomyces lincolnensis* wild-type strain and high-yield strain unraveled several mutations that contribute to lincomycin overproduction (Wang et al., 2020). Besides, Liu et al. identified 133 mutation sites through comparative genome analysis between *Streptomyces bingchenggensis* high-yield mutant strain BC04 and parental strain BC-101-4, and three targets were validated to be responsible for the overproduction of milbemycin A3/A4 (Liu Y. et al., 2021). Therefore, combined genome analysis with metabolic engineering has great potential to further improve the production of butenyl-spinosyn.

In addition to metabolic engineering, medium optimization is essential to maximize the production of secondary metabolites (Yu et al., 2024; Zhang et al., 2023). It is noteworthy that optimizing the components of the fermentation medium, such as carbon sources, nitrogen sources and inorganic salts, can significantly improve the yield of the engineered strains. In one study, *SLCG_4846* and *SLCG_2919* were combined deletions in *Streptomyces lincolnensis* resulting in a 29.1% increase in lincomycin production, and subsequent optimization of the seed medium further increased the yield by 55.1% (Cai et al., 2024). Meanwhile, by combining the overexpression of *asm13-17* and *asmUdpg* with a fructose feeding strategy, AP-3 production was increased by 2.35-fold (Du and Zhong, 2018). These findings demonstrate that optimizing the medium is a viable strategy to maximize the production potential of engineered strains.

In recent years, metabolomics analysis has been used to decipher the complex metabolic networks in diverse microorganisms (Liu P. et al., 2021; Rang et al., 2020). Moreover, metabolomics is commonly distinguished into targeted and untargeted metabolomics (Gorrochategui et al., 2016). Compared to untargeted metabolomics, targeted metabolomics enables accurate detection of metabolic bottlenecks through systematic analysis of metabolites closely related to target product biosynthesis, such as organic acids, amino acids, and cofactors. Owing to the prominent advantages of targeted metabolomic analysis, it has been successfully applied to deepen understanding of the mechanisms of secondary metabolite overproduction. For example, Liu et al. identified the critical role of methylmalonyl-CoA in the high-yield candicidin-producing strain with *AdpA* overexpression by targeted metabolomics analysis (Liu et al., 2019). Similarly, Yuan et al. exploited targeted metabolomics to investigate the metabolic regulatory mechanisms of erythromycin overproduction through ammonium sulfate feeding in *Saccharopolyspora erythraea* (Yuan et al., 2023). These cases confirm that targeted metabolomics offers a valuable technique to elucidate metabolic networks as well as underlying mechanisms of high-yield strains.

In the present work, five targets were selected for overexpression based on comparative genomic analysis. As a result, two of the targets, dTDP-glucose 4,6-dehydratase (*sp6746*) and NAD-glutamate dehydrogenase (*sp1322*), were identified as effective for improving butenyl-spinosyn yield. Then, combinatorial overexpression of *sp1322* and *sp6746* was conducted, yielding strain O1322-6746 with enhanced butenyl-spinosyn production. Moreover, aG6 and O1322-6746 were subject to comparative targeted metabolomic analysis to elucidate the high-yield mechanism of O1322-6746. Afterwards, single-factor experiments, Plackett-Burman design, steepest ascent method, and response surface methodology with central composite design (CCD) were utilized to optimize the fermentation medium of O1322-6746. Finally, the high yield of butenyl-spinosyn achieved in a 5-L bioreactor provided valuable insights for its industrial application.

## 2 Materials and methods

### 2.1 Strains, plasmids, and primers

All strains used in this study are described in Supplementary Table S1. *E. coli* DH5α and *E. coli* ET12567/pUZ8002 were used for plasmid construction and conjugative transfer between *E. coli* and *S. pogona*.

### 2.2 Plasmids and recombinant strains construction

The integrative vector pIB139 was used to generate recombinant plasmids for gene overexpression. The plasmids and primers used in this study are displayed in Supplementary Table S2. Using the genome of *S. pogona* aG6 as a template, the coding sequences of *sp2611*, *sp1943*, *sp6746*, *sp4102*, and *sp1322* were amplified by the primer pairs 2611F/2611R, 1943F/1943R, 6,746-F/6746-R, 4102-F/4102-R, and 1322-F/1322-R, respectively. Next, the segments were ligated into pIB139 and digested with *Eco*RI and *Nde*I to obtain recombinant plasmids. For the construction of recombinant vectors for co-overexpressing *sp1322* and *sp6746*, primers 6,746-F2 and 6,746-R were used to amplify the *sp6746-2* fragment. The *sp6746-2* and *sp1322* fragments were inserted into pIB139 vector by *Nde*I and *Eco*RI, thereby obtaining pIB1322-6746. Those recombinant plasmids were confirmed by DNA sequencing using pIB139YZ-F/pIB139YZ-R and then introduced into *S. pogona* through intergeneric conjugation (Supplementary Figure S1).

### 2.3 Culture conditions for *Saccharopolyspora pogona* and detection of butenyl-spinosyn

The culture medium for *S. pogona* is shown in Supplementary Table S3. The SP medium was chosen for spore inoculation. The ISP4 medium was used for intergeneric conjugation. The fermentation medium used for intracellular metabolite extraction is listed in Supplementary Table S3. For butenyl-spinosyn flask fermentation, *S. pogona* was cultivated in a seed medium for 72 h. Later, 1.5 mL seed broth was pipetted into 30 mL fermentation medium with a cultivation time of 5 days at 30°C. The fermentation was extracted with two volumes of methanol for overnight. Next, the sample was subjected to centrifugation at 5000 rpm for 10 min. The filtered supernatants were analyzed by HPLC. The samples were injected into C18 column (ZORBAX Eclipse XDE), butenyl-spinosyn was separated by acetonitrile: methanol: 0.05% ammonium acetate buffer at a 4.5:4.5:1 volumetric ratio. The flow rate was set at 1 mL/min with detection at 254 nm.

### 2.4 Cultivation in a 5-L bioreactor

Batch fermentation was performed in a 5-L bioreactor (Shanghai Guoqiang Bioengineering Equipment Co., Ltd.). The seed culture was prepared using the same conditions as in shake flask fermentation. Then 5% seed broth with 2.5 L fermentation medium was cultured at 30°C for 120 h. The bioreactor fermentation process parameters were set as follows: aeration rate at 3 vvm and impeller speed at 350 rpm. Online monitoring of changes in CER, OUR, and pH curves. Samples were collected every 12 h to monitor yield, biomass and residual glucose. Biomass was determined as follows: 10 mL fermentation broth was collected and weighed. The broth was separated by centrifugation at 5000 rpm for 10 min. After the supernatant was removed, the pellet was weighed. Biomass was calculated as the ratio of wet cell weight to medium weight.

### 2.5 Intracellular metabolites collection and extraction

The samples were harvested at 72 h. The intracellular metabolites extraction and determination were performed as reported previously (Yuan et al., 2023). 1 mL of the sample was taken and filtered through a vacuum filtration device to collect bacterial cells on the filter paper. Rinsed the filter paper three times with deionized water. Immediately transfer the filter paper to a pre-cooled 50 mL centrifuge tube containing liquid nitrogen. Then, add 100 μL of internal standard (IS) before sample extraction. The sample was mixed with 25 mL of 75% ethanol solution preheated to 95°C, followed by 30 s vortex oscillation to ensure uniform mixing of the sample, and then the mixture was heated in boiling water container for 10 min. The heated extract was centrifuged under 4°C. Then, we harvested the supernatant and passed it through a filter membrane. The filtered supernatant was concentrated with a vacuum evaporator, with vacuum pressure and rotation speed adjusted according to the solution volume. After complete evaporation, the residue was redissolved in ultrapure water to obtain 300 mg solution. The reconstituted solution was stored in a − 80°C freezer for 45 min, followed by overnight lyophilization for subsequent derivatization.

### 2.6 Sample derivatization

For intracellular amino acid derivatization, the freeze-dried sample was supplemented with 80 μL of acetonitrile as well as 80 μL of N-Methyl-N-(tert-butyldimethylsilyl) trifluoroacetamide (MTBSTFA), followed by incubation at 70°C for 2 h. The suspension was pelleted by centrifugation. Next, 160 μL of liquid fraction was withdrawn and passed through the 0.45 μm membrane. For organic acid and phosphate sugar derivatization, the samples were treated with 50 μL of methoxyammonium pyridine solution (20 mg/L) and kept at 70°C for 1 h. Following cooling to ambient temperature, 80 μL of N-Methyl-N-(trimethylsilyl) trifluoroacetamide/ Trimethylchlorosilane (MSTFA/TMSC) mixture (20:1, v/v) was pipetted. Subsequently, the derivatization was continued at 70°C for 1 h. After cooling for 10–15 min, the mixture was subjected to centrifugation. The liquid phase was separated by filtration before analysis.

### 2.7 Detection of intracellular metabolites

The intracellular metabolite detection was conducted as reported previously (Yuan et al., 2023). Gas chromatography–mass spectrometry (GC–MS) was used for intracellular metabolites determination. Chromatographic separation was performed on a HP5-5% phenylmethylsiloxane column, injector temperature of 250°C, and using helium as carrier gas (1 mL/min). The mass spectrometry conditions were: electron bombardment of the ion source (70 eV), selection of ion monitoring (SIM) mode with temperatures set at: transmission circuit (280°C), ionizer (230°C), and mass analyzer (150°C). The heating program for different types of metabolites was as follows: the starting temperature for the analysis of organic acids, phosphorylated sugars, and sugar alcohols was 70°C and held for 60 s, then ramped to 300°C at a temperature gradient of 10°C/min with a dwell time of 10 min; Initial temperature for amino acid analysis was 100°C and kept at 60s, then heated to 300°C with a temperature gradient of 10°C/min and was held over 10 min. Data collection and processing were carried out using MSD ChemStation software. Intracellular metabolite concentrations were quantified using isotope dilution mass spectrometry (IDMS).

### 2.8 Statistical analysis

Three biological triplicates were executed for all experiments. All data were presented as mean ± SD. Statistical analyses were performed with Student’s *t*-test by GraphPad Prism 5.0 and *p* < 0.05 indicates statistical significance.

## 3 Results

### 3.1 Comparative genomics identified mutated genes in genome of aG6

**Figure 1:**
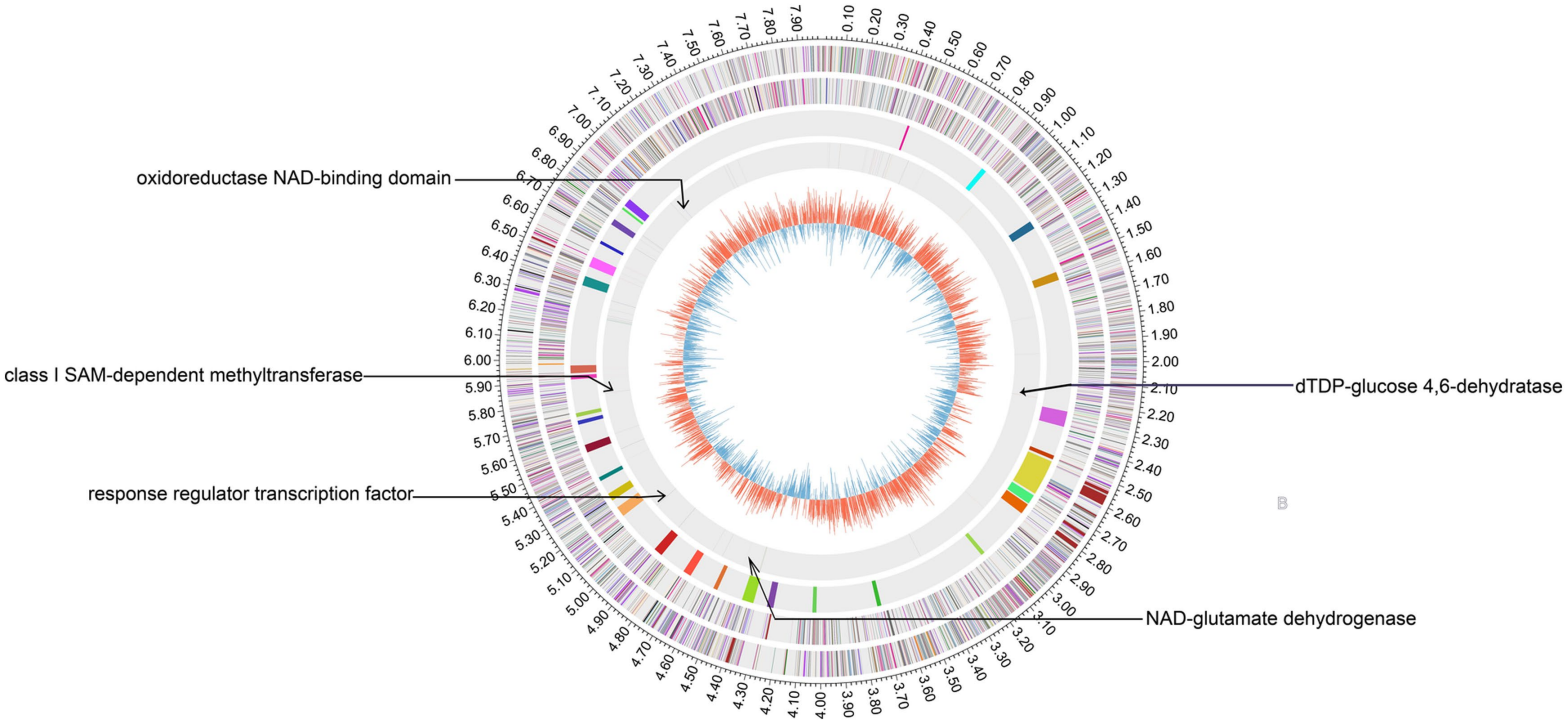
The chromosomal map of genetic variation between *S. pogona* WT and aG6. From the outermost to the innermost circles: Circles 1 and 2: Predicted protein-coding sequences on the forward and reverse strands, respectively, colored according to COG (Clusters of Orthologous Groups) functional categories; Circle 3: Distribution of secondary metabolite gene clusters; Circle 4: Genomic variations between aG6 and WT; circle 5:GC content. Five validated genes are highlighted with arrows.

**Figure 2:**
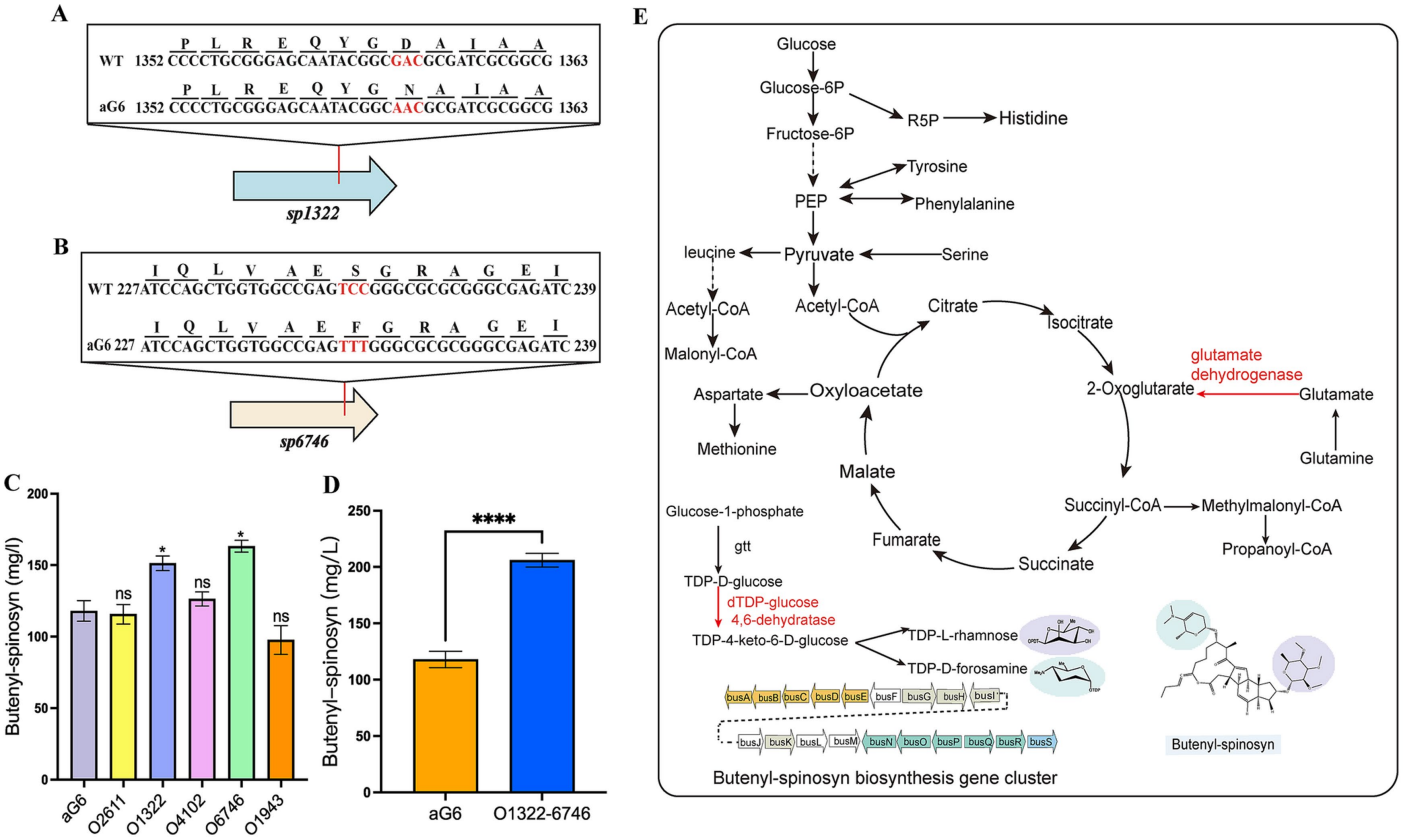
Metabolic engineering of *S. pogona* for the production of butenyl-spinosyn. **(A,B)** Represent DNA and deduced protein variation between WT and aG6 of *sp1322* and *sp6746*, respectively. Mutated sites are marked in red. **(C)** Butenyl-spinosyn production of aG6 and overexpression mutants. **(D)** Butenyl-spinosyn production of aG6 and O1322-6746. **(E)** Simplified diagram of butenyl-spinosyn biosynthesis in O1322-6746. For **(C,D)**, the data were obtained from three independent experiments. Differences were analyzed by Student’s *t*-test. ****p ≤ 0.0001, ***p < 0.001, **p < 0.01, *p < 0.05, and ns, not significant.

In our previous study, the whole genomes of WT and aG6 strains were sequenced and comparatively analyzed (Zhao et al., 2024). Comparative analysis identified 17 nonsynonymous mutations and 18 frameshift mutations between WT and aG6. Both nonsynonymous and frameshift mutations affect protein structure and function. Nonsynonymous mutations alter amino acid sequences and potentially influence cellular physiological processes, whereas frameshift mutations result in complete changes of subsequent codon sequences from the mutation point (Li et al., 2013). Butenyl-spinosyn biosynthesis is regulated by complex mechanisms, such as primary metabolism, secondary metabolism, and transcriptional factors. Among the genes with nonsynonymous mutations and frameshift mutations, we selected five genes that are responsible for encoding primary metabolism, secondary metabolism, or transcription factors. The positions of these five genes in the genome are shown in Figure 1. These genes encode diverse functional proteins, including *sp2611* (G6.63.6_GM002611) encoding class I SAM-dependent methyltransferase, *sp1943* (G6.63.6_GM001943) encoding response regulator transcription factor, *sp6746* (G6.63.6_GM006746) encoding dTDP-glucose 4,6-dehydratase, *sp4102* (G6.63.6_GM004102) encoding oxidoreductase NAD-binding domain, and *sp1322* (G6.63.6_GM001322) encoding glutamate dehydrogenase. The corresponding mutants were named O2611, O1943, O6746, O4102, and O1322, respectively. As shown in Figure 2C, the overexpression of *sp1322* boosted the butenyl-spinosyn yield by 27.5%. The mutation sites of *sp1322* are shown in Figure 2A, in which GAC in position 1,359–1,361 was substituted with AAC in aG6, leading to an aspartic acid to asparagine mutation. Additionally, overexpression of *sp6746* resulted in a 30.8% improvement in butenyl-spinosyn yield (Figure 2C). The mutation sites of *sp6746* are shown in Figure 2B, where TCC in position 233–235 was replaced with TTT in aG6, causing a serine to phenylalanine substitution. On the contrary, overexpression of the other three mutated genes had no remarkable effect on the yield of butenyl-spinosyn.

### 3.2 Combinatorial overexpression of sp1322 and sp6746 enhanced butenyl-spinosyn production

Glutamate dehydrogenase is a key metabolic enzyme that catalyzes the reaction of glutamate to *α*-ketoglutarate while generating ammonia and NAD(P)H. Therefore, this enzyme plays a key role in amino acid metabolism and energy production (Liu Y. et al., 2021). In addition to central metabolism, sugar modification also plays a vital role in natural product biosynthesis. In the biosynthesis of butenyl-spinosyn, forosamine and tri-*O*-methyl rhamnose are individually attached to the aglycone via dTDP-forosamine and dTDP-rhamnose, respectively (Hahn et al., 2006). The dTDP-glucose 4,6-dehydratase catalyzes the conversion of TDP-D-glucose to TDP-4-keto-6-deoxy-D-glucose, which then functions as a precursor for dTDP-rhamnose and dTDP-forosamine biosynthesis (Pan et al., 2011). The positions of *sp1322* and *sp6746* within the pathway are illustrated in Figure 2E. To determine whether the combined overexpression of *sp1322* and *sp6746* could enhance the yield of butenyl-spinosyn, strain O1322-6746 with co-overexpression of *sp1322* and *sp6746* was constructed. The results indicated that strain O1322-6746 achieved higher butenyl-spinosyn yield than strains with individual overexpression of *sp1322* or *sp6746*, showing a 76.9% increase compared to aG6 (Figure 2D).

### 3.3 Validation of butenyl-spinosyn production by O1322-6746 in a 5-L bioreactor

**Figure 3:**
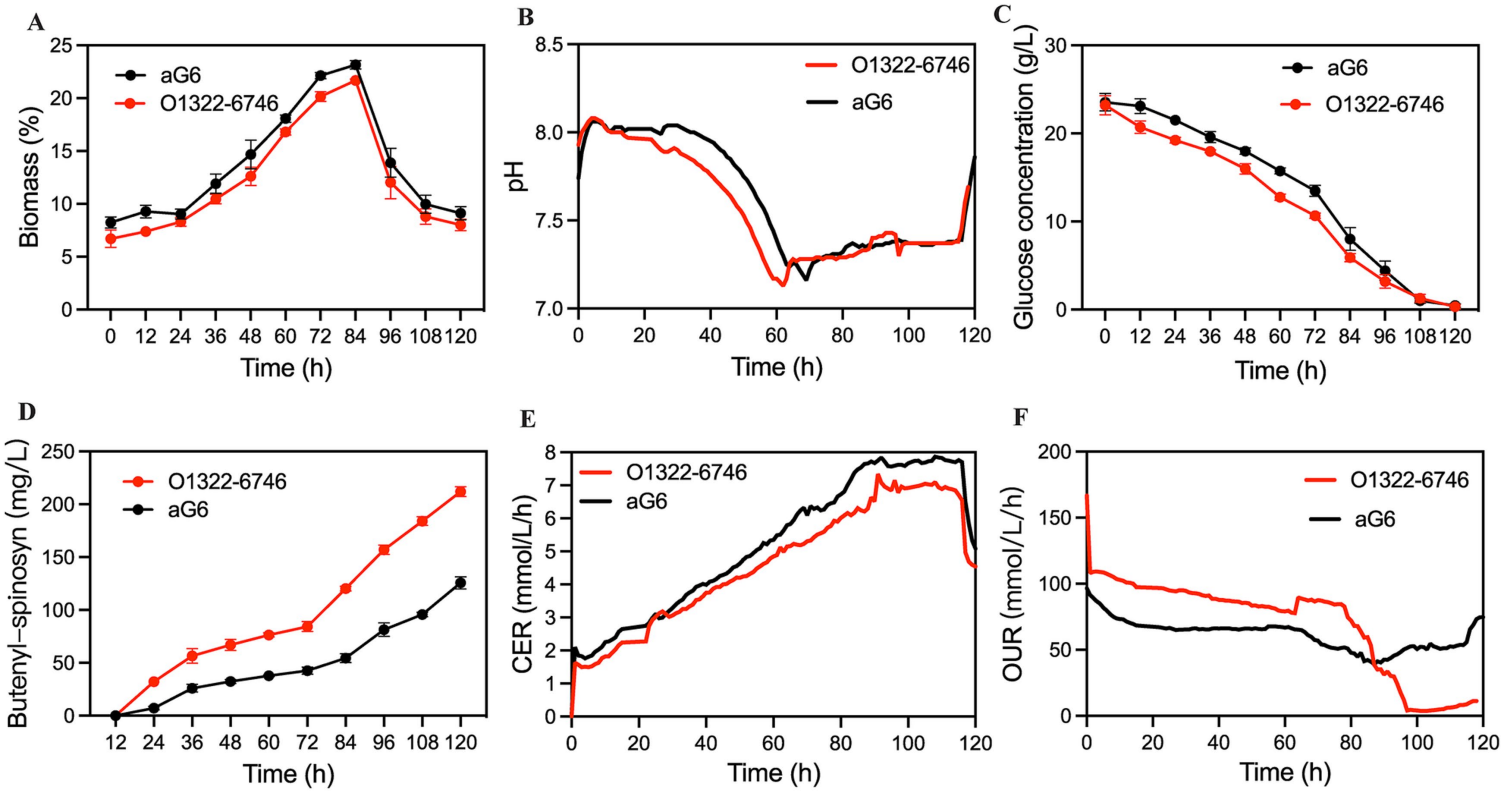
Comparison of fermentation parameters between O1322-6746 and aG6 in a 5-L bioreactor. **(A)** Biomass. **(B)** pH. **(C)** Residual sugar. **(D)** Butenyl-spinosyn production. **(E)** CER. **(F)** OUR.

To verify the yield stability of O1322-6746, fermentation was performed in a 5-L bioreactor. As depicted in Figure 3A, the overall trend of biomass between aG6 and O1322-6746 was similar. After 20 h of cultivation, both strains exhibited a significant increase in biomass. Notably, strain aG6 exhibited a slightly elevated growth rate relative to O1322-6746 during this phase. The biomass of both strains peaked at 80 h, with strain aG6 reaching 23.15% and O1322-6746 attaining 21.68%. During fermentation, the biomass of aG6 was mildly higher than that of O1322-6746, indicating that gene overexpression might have an impact on strain growth, but the overall effect was minimal. The carbon dioxide evolution rate (CER) parameters further corroborated this trend and demonstrated that gene overexpression had a relatively subtle influence on respiratory metabolism. As shown in Figure 3E, during the early fermentation stage, aG6 and O1322-6746 maintained similar respiratory intensity. However, upon entering the exponential growth phase, the CER peak of O1322-6746 was lower than that of aG6. The change in pH of fermentation broth could reflect the metabolic state to a certain extent. Due to the faster glucose consumption rate of O1322-6746 compared to aG6 (Figure 3C), O1322-6746 showed an earlier decline in fermentation broth pH during the initial stages of fermentation (Figure 3B). At the end of fermentation, the yield of butenyl-spinosyn from O1322-6746 was 212.6 mg/L, representing a 77.1% improvement over aG6 (Figure 3D).

### 3.4 Comparative targeted metabolomic analysis of aG6 and O1322-6746

To elucidate the physiological metabolic mechanisms underlying the enhanced butenyl-spinosyn production in O1322-6746, comparative targeted metabolomic analysis of aG6 and O1322-6746 was performed using isotope-assisted GC-IDMS method. A total of 32 metabolites were measured, including 17 amino acids, 10 phosphorylated sugars, and 5 organic acids.

#### 3.4.1 Amino acid analysis

**Figure 4:**
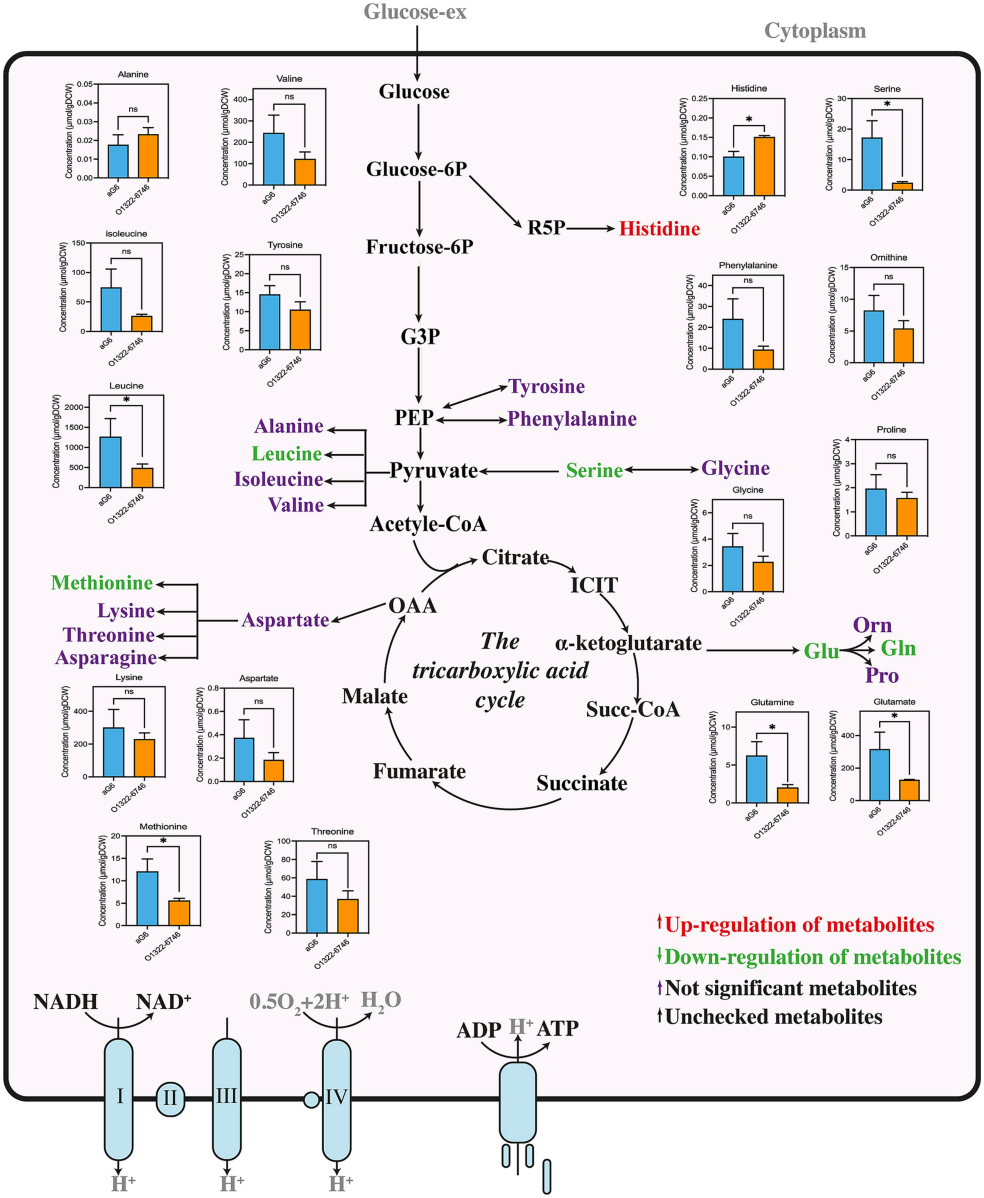
Intracellular amino acid metabolic network in aG6 and O1322-6746. All data are given as mean ± SD (*n* = 3). Statistical analysis was performed using Student’s *t*-test. *p < 0.05 and ns, not significant.

The intracellular amino acid analysis of aG6 and O1322-6746 are shown in Figure 4. Interestingly, both glutamate and glutamine showed a similar downward trend. This phenomenon could be explained by glutamate dehydrogenase overexpression, which facilitated the conversion of glutamate to *α*-ketoglutarate, and led to a striking decrease in the levels of glutamate and glutamine. In addition, the pool size of methionine in O1322-6746 was distinctly smaller than that in aG6. This reduction could be explained by dTDP-glucose 4,6-dehydratase overexpression, which promoted the biosynthesis of rhamnose, thereby enhancing SAM-dependent methylation processes (Xue et al., 2013). Consequently, these augmented processes likely facilitated methionine degradation, resulting in reduced methionine levels. Serine can be converted to acetyl-CoA through pyruvate, which serves as a key precursor in various metabolic pathways. The reduction in serine concentration was hypothesized to stimulate the transition of serine to pyruvate, subsequently to acetyl-CoA (Liu et al., 2018), thus favorably supplying the precursors necessary for the biosynthesis of butenyl-spinosyn. There was a significant increase in the content of histidine in O1322-6746. The biosynthesis of histidine begins with 5-phosphoribosyl-1-pyrophosphate (PRPP), a key product of the pentose phosphate pathway. It was speculated that changes in the pentose phosphate pathway affected the biosynthesis and metabolism of histidine in the O1322-6746. Furthermore, a notable decrease in the concentration of leucine was observed in the O1322-6746. The biosynthesis of leucine requires precursors and cofactors, including pyruvate, acetyl-CoA, and NADPH (Liu et al., 2019). Therefore, we postulated that the striking decrease in leucine guaranteed adequate pyruvate, acetyl-CoA, and NADPH for the biosynthesis of butenyl-spinosyn in O1322-6746.

#### 3.4.2 Phosphate sugars and organic acids analysis

**Figure 5:**
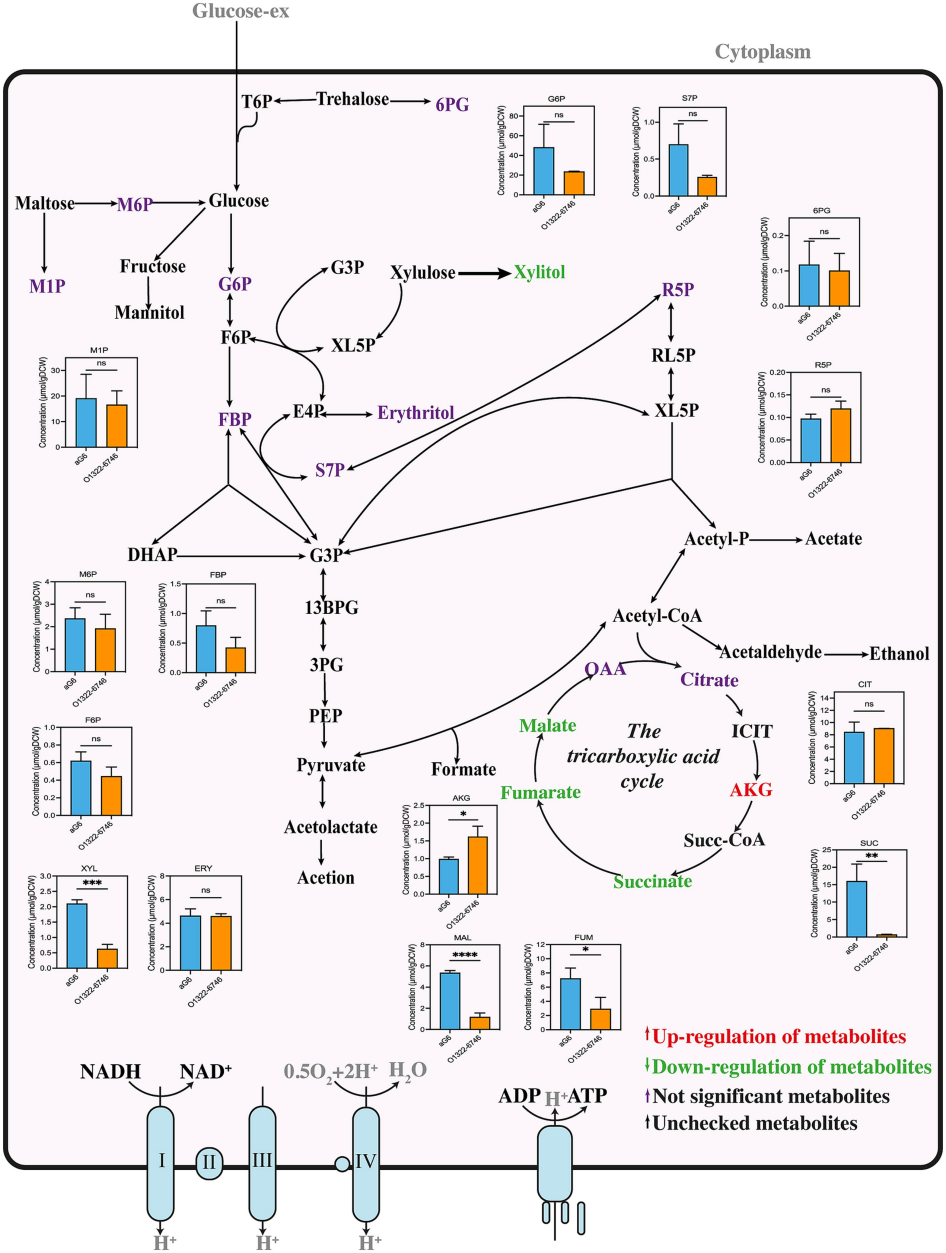
Intracellular phosphosugar and organic acids metabolic network in aG6 and O1322-6746. All data are given as mean ± SD (*n* = 3). Statistical analysis was performed using Student’s *t*-test. The levels of significance are ****p ≤ 0.0001, ***p < 0.001, **p < 0.01 and *p < 0.05; ‘ns’ means no significant.

Besides amino acids, phosphate sugars and organic acids in aG6 and O1322-6746 were detected and illustrated in Figure 5. Firstly, we found that the concentrations of intermediate metabolites in the tricarboxylic acid (TCA) cycle including malate, fumarate and succinate, were distinctively decreased in O1322-6746. On the contrary, the *α*-ketoglutarate content was greater in O1322-6746 than in aG6, suggesting that the overexpression of glutamate dehydrogenase expedited the conversion of glutamate to α-ketoglutarate. Additionally, the xylitol content was significantly decreased in O1322-6746. This decrease was likely due to xylitol entering glycolysis via the pentose phosphate pathway. Consequently, accelerated glucose consumption in O1322-6746 indirectly facilitated xylitol metabolism.

### 3.5 Optimized medium for O1322-6746 to improve butenyl-spinosyn yield

To take full advantage of the high-yield potential of the O1322-6746, culture medium optimization is essential. This study optimized the culture medium for O1322-6746 through single-factor experiments, Plackett-Burman design, steepest ascent method, and response surface methodology with central composite design (CCD).

#### 3.5.1 Single-factor experiments

**Figure 6:**
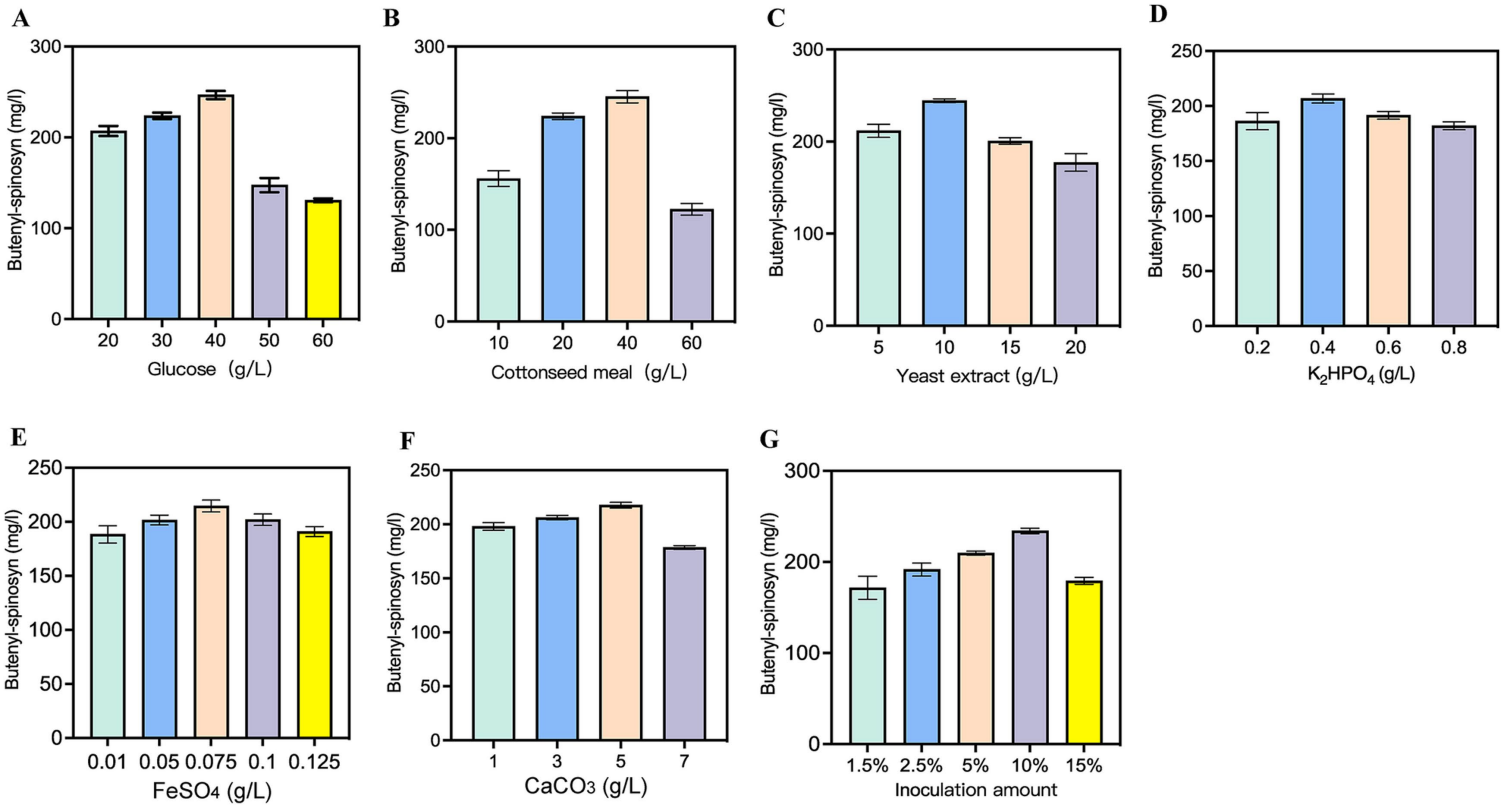
Effect of fermentation conditions on butenyl-spinosyn production. **(A)** Glucose. **(B)** Cottonseed meal. **(C)** Yeast extract. **(D)** K_2_HPO_4_. **(E)** FeSO_4_. **(F)** CaCO_3_. **(G)** Inoculation amount.

Single-factor experiments were carried out to optimize the medium composition. Firstly, glucose concentration varied from 20 to 60 g/L, and the maximum butenyl-spinosyn production occurred at 40 g/L (Figure 6A). Subsequently, we optimized two nitrogen sources: cottonseed meal and yeast extract. For cottonseed meal, the levels ranging from 10 to 60 g/L were investigated, with the highest butenyl-spinosyn yield achieved at 40 g/L (Figure 6B). Similarly, when examining yeast extract at concentrations from 5 to 20 g/L, the optimal butenyl-spinosyn production was observed at 10 g/L (Figure 6C). Following this, we proceeded to optimize the concentrations of inorganic ions. For K₂HPO₄, the contents varying from 0.2 to 0.8 g/L were tested, resulting in a slight increase in butenyl-spinosyn production at 0.4 g/L (Figure 6D). For FeSO₄, the concentrations of 0.01 to 0.125 g/L were evaluated, with the marginally higher butenyl-spinosyn production achieved at 0.075 g/L (Figure 6E). Given that CaCO₃ could neutralize acids and maintain pH stability during fermentation, various CaCO₃ concentrations were tested. The results revealed that 5 g/L CaCO₃ yielded a moderate increase in butenyl-spinosyn production (Figure 6F). It has been reported that appropriate inoculum levels could accelerate microbial acclimatization during fermentation (Zhang et al., 2023). Herein, five inoculum concentrations (1.5, 2.5, 5, 10, and 15%) were investigated to optimize the inoculum amount. The butenyl-spinosyn yield increased gradually with the inoculum concentrations up to 10%, whereas a decline was observed at 15% (Figure 6G). This decrease was most likely due to insufficient dissolved oxygen resulting from excessive inoculum concentration.

#### 3.5.2 Plackett-Burman design and results

The Plackett-Burman design was established based on the single-factor experimental results (Supplementary Table S4). Among the 12 experimental groups, butenyl-spinosyn production exhibited significant variation extending from 96.1 mg/L to 248.3 mg/L (Supplementary Table S5). A multiple linear regression equation was developed from the Plackett-Burman design to characterize the relationship between variables. The model was presented as: Y=173.5+46.83A+15.0B+11.0C−3.33D+0.67E−0.67F+3.17G Besides, the results of the analysis of variance (ANOVA) of the Plackett-Burman test are listed in Supplementary Table S6. According to the ANOVA results, glucose, cottonseed meal, and yeast extract were identified to significantly impact butenyl-spinosyn production, with the *p*-values below 0.05. The *R*^2^ value of 0.9835 demonstrated that the model was able to explain about 98% of empirical data, with statistical significance (*p* = 0.0021).

#### 3.5.3 Three-factor steepest ascent experiment

Plackett-Burman analysis indicated that glucose, cottonseed meal, and yeast extract exhibited positive effects on butenyl-spinosyn yield. Therefore, the steepest ascent experiment was conducted with incremental concentrations of glucose, cottonseed meal, and yeast extract to enhance butenyl-spinosyn production. As shown in Supplementary Table S7, the maximum production of butenyl-spinosyn was found in the second group of experiments, which was near the optimum response area and selected for further optimization.

#### 3.5.4 CCD and response surface analysis

Next, CCD was used to optimize the glucose, cottonseed meal, and yeast extract concentrations. A series of 19 experiments under diverse designs were shown in Supplementary Tables S8, S9. Design-Expert 10.0 was used to evaluate the experimental data of CCD, and established a mathematical model: $\begin{array}{l} Y=292.31-5.66\;A+2.67\;B+2.08\;C+1.38\;AB-2.62\;AC+8.63BC\\ -9.84A^{2}-11.97B^{2}-8.78C^{2}. \end{array}$

**Figure 7:**
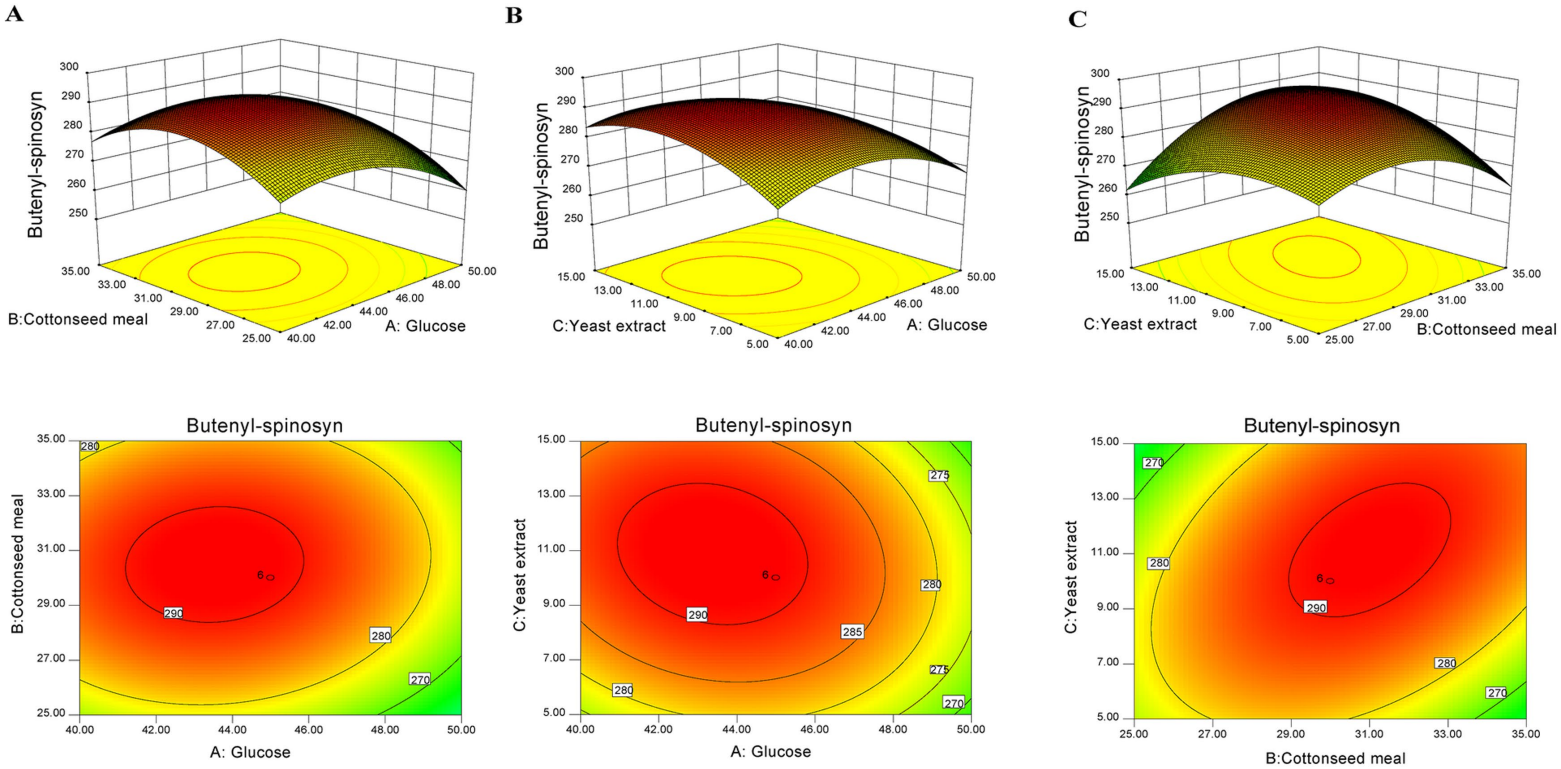
Response surface and two-dimensional contour map for butenyl-spinosyn production in O1322-6746 strain. **(A)** Interaction between glucose and cottonseed meal. **(B)** Interaction between glucose and yeast extract. **(C)** Interaction between cottonseed meal and yeast extract.

As shown in Supplementary Table S10, the *p*-value of the response surface regression model was under 0.0001 while the lack of fit *p*-value was above 0.05, indicating that the model was significant while the lack of fit was not notable. Meanwhile, the *R*^2^ and Adj-*R*^2^ were 0.9865 and 0.9731, respectively, which demonstrated that the model matched closely with the experimental data (Supplementary Table S11). The variance analysis of the model revealed that glucose, cottonseed meal and yeast extract had obvious effects on the butenyl-spinosyn yield, with significant interactive effects observed between glucose and yeast extract, as well as between cottonseed meal and yeast extract (Supplementary Table S10). The 3D response surface and contour plots were shown to illustrate the interplay between variables (Figure 7). Based on the model optimization, the maximum butenyl-spinosyn yield was predicted to be 293.7 mg/L at 43.45 g/L glucose, 30.93 g/L cottonseed meal and 11.28 g/L yeast extract.

### 3.6 5-L bioreactor validation yield of O1322-6746 with optimized medium

**Figure 8:**
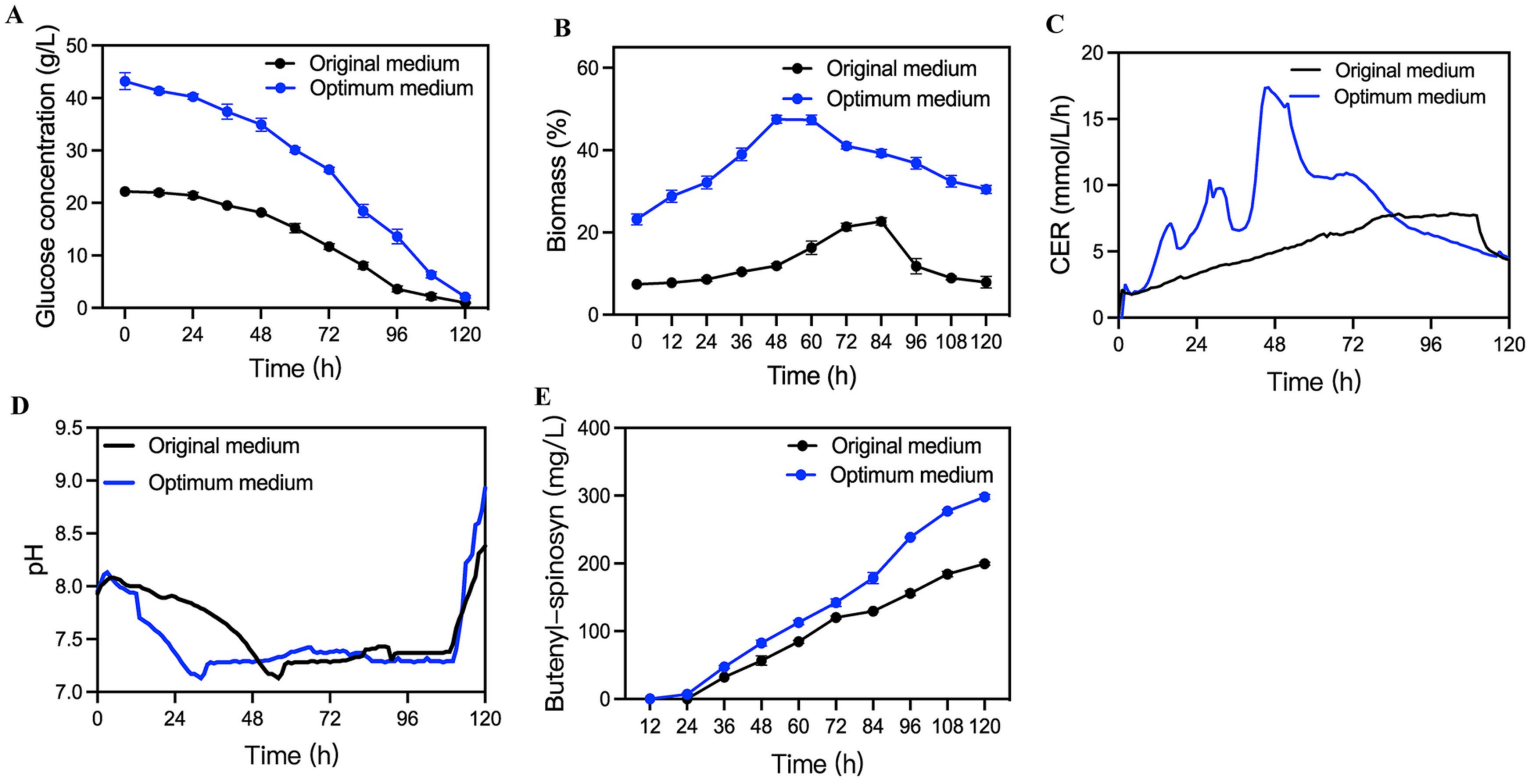
Time-course profiles of O1322-6746 fermentation in the optimized and initial medium in a 5-L bioreactor. **(A)** Residual sugar. **(B)** Biomass. **(C)** CER. **(D)** pH. **(E)** Butenyl-spinosyn production.

To assess the accuracy of the model, cultivation of O1322-6746 in the optimized medium was performed in a 5-L bioreactor. In the optimized medium, butenyl-spinosyn production by O1322-6746 reached 298.5 mg/L, 40.5% higher than that in the original medium (Figure 8E). Additionally, important fermentation parameters, including CER, OUR, pH (Figure 8D), biomass and residual sugars, were determined. As shown in Figure 8C, the peak CER values were observed at 84 h and 48 h in the original and optimized medium, respectively. The maximum CER values were 7.84 and 17.38 mmol/L/h in the original and optimized medium, respectively. The shortened time to peak CER and raised peak CER value indicated that the optimized medium provided more favorable conditions for the respiratory metabolism of O1322-6746. Moreover, the biomass of O1322-6746 in the optimized medium was superior to that in the original medium throughout the fermentation process. As shown in Figure 8B, the maximum biomass in the optimized medium was 47.5%, which was a 2.09-fold enhancement compared to the original medium (the maximum biomass value in the original medium was 22.6%). This increase in biomass might be attributed to the rational ratio of medium components in the optimized medium. Moreover, this observation was aligned with the higher glucose utilization rate of O1322-6746 in the optimized medium (Figure 8A). Thereby we successfully optimized the fermentation medium which was more beneficial for the proliferation of O1322-6746.

## 4 Discussion

Actinomycetes produce diverse secondary metabolites, including pesticides, antibiotics and immunosuppressive compounds, which are extensively applied in therapeutics, agronomy and related fields (Palazzotto et al., 2019). Nevertheless, secondary metabolite yields in native hosts remain much below the needs of large-scale fermentation, owing to their non-essential role and metabolic overhead (Bu et al., 2021). To address this issue, the combination of strategies has been proven to be more efficient in secondary metabolites production improvement (Du et al., 2017; Cai et al., 2021; Yin et al., 2021). In this study, metabolic engineering combined with fermentation optimization was employed in *S. pogona* and resulted in enhanced butenyl-spinosyn production.

In the past several decades, random mutagenesis has been widely used to improve secondary metabolite production, having not only enhanced metabolite yields but also generated valuable resources for subsequent investigation (Du et al., 2017). Besides, the development of genome sequencing has enabled systematic analysis of mutations in high-producing strains derived from random mutagenesis (Xie et al., 2019; Li et al., 2022). Moreover, the analyzed mutation sites from comparative genome analysis offer meaningful insights into metabolic engineering strategies for the enhancement of strain productivity (Zhang et al., 2017). In this work, we verified two effective mutated sites (dTDP-glucose 4,6-dehydratase and NAD-glutamate dehydrogenase) for yield improvement of butenyl-spinosyn based on our previous comparative omics analysis.

During the process of secondary metabolite biosynthesis, supplying adequate precursors such as sugar moieties, methylmalonyl-CoA, and acyl-CoA is indispensable (Li et al., 2021a). Notably, primary metabolism acts as the main source of those precursors (Li et al., 2021b). Therefore, it is essential to optimize the precursor availability. The biosynthetic pathway of rhamnose and forosamine plays a crucial role in butenyl-spinosyn formation (Hong et al., 2006). In particular, rhamnose is not only a glycosyl group in butenyl-spinosyn but also essential for primary metabolism (Pan et al., 2011). The common intermediate for 3-O-methylrhamnose and forosamine biosynthesis of butenyl-spinosyn is TDP-4-keto-6-deoxy-D-glucose. The dTDP-glucose 4,6-dehydratase encoded by *sp6746* is involved in the conversion step from glucose-1-phosphate to TDP-4-keto-6-deoxy-D-glucose. Hence, the overexpression of *sp6746* was speculated to enhance the metabolic flux of glucose-1-phosphate toward the TDP-4-keto-6-deoxy-D-glucose, ultimately promoting the biosynthesis of both sugar moieties. It is known that succinyl-CoA either participates in the TCA cycle via succinyl-CoA dehydrogenase oxidation or undergoes reversible isomerization to methylmalonyl-CoA (Ke et al., 2022). In our work, glutamate dehydrogenase overexpression might have stimulated the metabolic flux of succinyl-CoA toward direct precursor pathways of butenyl-spinosyn, thereby promoting its biosynthesis. This also suggests that modulating indirect precursor flux through key enzyme manipulation benefits the accumulation of direct precursors of target secondary metabolites.

Metabolic engineering combining multiple targets is a promising approach for increasing the yield of secondary metabolites. Co-overexpression of *asm13-17* and *asm Udpg* in *Actinosynnema pretiosum* led to an improvement in AP-3 production (Du et al., 2017). The deletion of the repressor and overexpression of the activator resulted in an increase in thaxtomin production (Li Z. et al., 2021). In our study, combinatorial overexpression of *sp1322* and *sp6746* further improved the production of butenyl-spinosyn. This observation demonstrates that combinatorial optimization of primary metabolism-related gene expression levels could enhance secondary metabolite yields.

The productivity of secondary metabolites is affected by fermentation medium (Yu and Wang, 2017). Consequently, we conducted fermentation optimization in *S. pogona* to further increase the butenyl-spinosyn. There are diverse methods for optimizing fermentation medium, which are used to provide nutrients and promote the growth and reproduction of cells (Shu et al., 2024). Firstly, the single-factor experiments are simple and easy to perform, with experimental design and data analysis being relatively straightforward (Yu et al., 2019). Following initial optimization through single-factor experiments, Plackett-Burman design enables the identification of significant variables. Subsequently, CCD and response surface methodology were used to analyze the interactions among these factors, establish a response surface model, and determine the optimal operating conditions. Overall, the application of those systematic medium optimization strategies has led to significant yield improvements for numerous secondary metabolites (Zheng et al., 2022; Hu et al., 2024). In this work, single-factor experiments, Plackett-Burman design, CCD and response surface methodology were systematically implemented to optimize the fermentation medium of the O1322-6746 and validated in a 5-L bioreactor. The butenyl-spinosyn yield of O1322-6746 reached 298.5 mg/L, which was higher than other reports.

## 5 Conclusion

In this work, two targets (glutamate dehydrogenase (*sp132*2) and dTDP-glucose 4,6-dehydratase (*sp6746*)) were identified based on comparative genomic analysis. The hyper-producing engineering strain O1322-6746 was obtained by combinatorial overexpression of *sp1322* and *sp6746*. Next, a comparative targeted metabolomic analysis of strains aG6 and O1322-6746 was performed to investigate the correlation between butenyl-spinosyn biosynthesis and the primary metabolic network. Moreover, by optimizing the fermentation medium, the butenyl-spinosyn production of O1322-6746 reached 298.5 mg/L in a 5-L bioreactor, which is the highest ever reported. The results of our work provide a feasible strategy for improving the production of butenyl-spinosyn and other secondary metabolites.

## Data Availability

The datasets presented in this study can be found in online repositories. The names of the repository/repositories and accession number(s) can be found in the article/Supplementary material.
