# Supplementary material for: Improving butenyl-spinosyn production in Saccharopolyspora pogona through combination of metabolic engineering and medium optimization
Source: Front Microbiol. 2025 Apr 23;16:1561042. doi: 10.3389/fmicb.2025.1561042 (PMC12055833; doi:10.3389/fmicb.2025.1561042)
Supplement: Supplementary file 1 [file Supplementary_file_1.docx]

**Supporting information**

**Improving butenyl-spinosyn production in *Saccharopolyspora pogona* through combination of metabolic engineering and medium optimization**


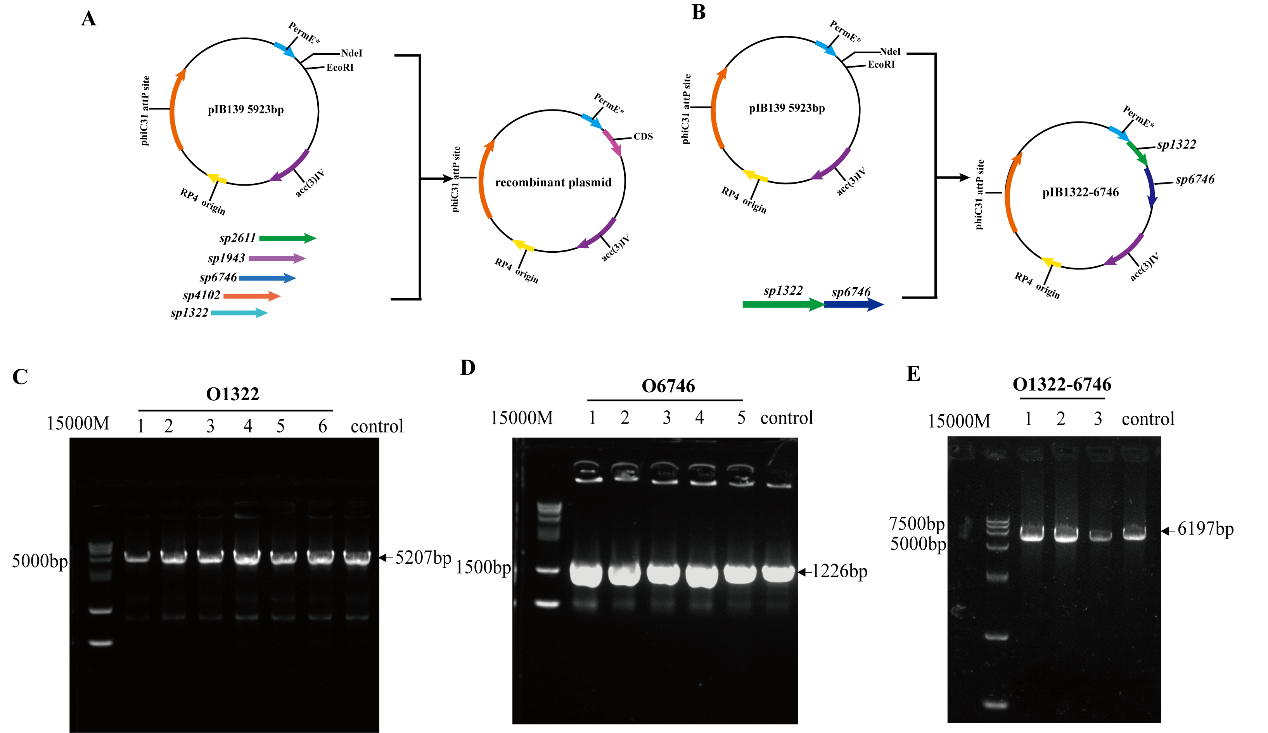


**Fig. S1** Verification of targets overexpression in the recombinant strain. (A) Construction process of individual gene overexpression; (B) Construction process of co-overexpression plasmid for *sp1322* and *sp6746*; (C) Verification of O1322 by PCR ampliﬁcation. Line1-6: the transformants of O1322, Line 7: pIB1322 as the template; (D) Verification of O6746 by PCR ampliﬁcation. Line 1-5: the transformants of O6746, Line 6: pIB6746 as the template; (E) Verification of O1322-6746 by PCR ampliﬁcation. Line 1-3: the transformants of O1322-6746, Line 4: pIB1322-6746 as the template.

Table S1 Strains and plasmids used in this study

| Strains or plasmids | Descriptions | Sources or references |
| --- | --- | --- |
| strains |  |  |
| aG6 | Mutagenesis of High-Yielding Strains of *S. pogona* | ^1^ |
| O2611 | aG6 harboring pIB2611 plasmid, for the overexpression of *sp2611* | This study |
| O1943 | aG6 harboring pIB1943 plasmid, for the overexpression of *sp1943* | This study |
| O6746 | aG6 harboring pIB6746 plasmid, for the overexpression of *sp6746* | This study |
| O4102 | aG6 harboring pIB4102 plasmid, for the overexpression of *sp4102* | This study |
| O1322 | aG6 harboring pIB1322 plasmid, for the overexpression of *sp1322* | This study |
| O1322-6746 | aG6 harboring pIB1322-6746 plasmid, for the co-overexpression of *sp1322* and *sp6746* | This study |
| Plasmids |  |  |
| pIB139 | Integrative vector between *E. coli* and *Streptomyces,* containing *ermE**p promoter | Lab store |
| pIB2611 | pIB139 harboring *sp2611* under control of *PermE** | This study |
| pIB1943 | pIB139 harboring *sp1943* under control of *PermE** | This study |
| pIB 6746 | pIB139 harboring *sp6746* under control of *PermE** | This study |
| pIB4102 | pIB139 harboring *sp4102* under control of *PermE** | This study |
| pIB1322 | pIB139 harboring *sp1322* under control of *PermE** | This study |
| pIB1322-6746 | pIB139 harboring *sp1322* and *sp6746* under control of *PermE** | This study |

**Table S2** Primers used in this study

| Name | Sequence (5**′-**3**′**) (restriction site underlined) | Use |
| --- | --- | --- |
| 2611F | GTGCCGGTTGGTAGGATCCACATATGGTGAACAGCGAAGGAACCGTG | Amplification of G6.63.6_GM002611 for the construction of pIB2611 |
| 2611R | AACAGCTATGACATGATTACGAATTCTCAGTTCGTTTGGTGTCGGC |  |
| 1943F | GTGCCGGTTGGTAGGATCCACATATGATGGCCCCGCACCCGCGGG | Amplification of G6.63.6_GM002611 for the construction of pIB1943 |
| 1943R | AACAGCTATGACATGATTACGAATTCTCATCGGATCACGCCGGCC |  |
| 1322-F | GTGCCGGTTGGTAGGATCCACATATGATGACCTCGAACCCCGGACAG | Amplification of G6.63.6_GM001322 for the construction of pIB1322 |
| 1322-R | AACAGCTATGACATGATTACGAATTCTCACCGGACCATGCTGCGC |  |
| 4102-F | GTGCCGGTTGGTAGGATCCACATATGTTGGCGCTGGGGGATCTGG | Amplification of G6.63.6_GM004102 for the construction of pIB4102 |
| 4102-R | AACAGCTATGACATGATTACGAATTCTCAAAATAGGCTAAGCGGCATTGAGG |  |
| 6746-F | GTGCCGGTTGGTAGGATCCACATATGATGCGGATTCTGGTCACCGGC | Amplification of G6.63.6_GM006746 for the construction of pIB6746 |
| 6746-R | AACAGCTATGACATGATTACGAATTCTCATCGCGCCACCGCCGAT |  |
| pIB139YZ-F | TGCTAGTCGCGGTTGATCG | Primers for verification of pIB plasmid construction |
| pIB139YZ-R | TATGCTTCCGGCTCGTATGT |  |
| 6746-F2 | TGCGCAGCATGGTCCGGTGAATGCGGATTCTGGTCACCGGC | Amplification of G6.63.6_GM006746 for the construction of pIB1322-6746 |

**Table S3** Culture medium of *S. pogona* used in this study

| Media name | Composition |
| --- | --- |
| SP medium | glucose 10g/L, soluble starch 10 g/L, yeast extract 5 g/L, N-Z-Amine 5 g/L, CaCO_3_ 1 g/L, agar 20 g/L, pH 7.5 |
| Seed medium | glucose 10 g/L，MgSO_4_·7H_2_O 2 g/L, KH_2_PO_4_ 0.5 g/L，yeast extract 30 g/L，peptonized milk 30 g/L, pH 7.5 |
| Fermentation medium | glucose 20 g/L, cottonseed meal 20 g/L, yeast extract 5 g/L, NaCl 3 g/L, K_2_HPO_4_ 0.4 g/L, FeSO_4_ .7H_2_O 0.05 g/L, CaCO_3_ 1 g/L, pH 7.2 |
| ISP4 medium | soluble starch 10.0 g/L, K_2_HPO_4_ 1.0 g/L, MgSO_4_·7H_2_O 1.0 g/L, NaCl 1.0 g/L, (NH_4_)_2_SO_4_ 2.0 g/L, CaCO_3_ 2.0 g/L, FeSO_4_·7H_2_O 0.001 g/L, MnCl_2_·4H_2_O 0.001 g/L, ZnSO_4_·7H_2_O 0.001 g/L, agar 20.0 g/L, pH 7.2 |
| Fermentation medium used for intracellular metabolite extraction | glucose 10 g/L, tryptone 30 g/L, yeast extract 3 g/L, MgSO_4_·7H_2_O 2 g/L, KH_2_PO_4_ 0.5 g/L, pH 7.5 |

**Table S4** Factors and levels in Plackett-Burman design

| Number | Variables | Experimental Levels | |
| --- | --- | --- | --- |
|  |  | -1 | +1 |
| A | Glucose | 40 | 60 |
| B | Cottonseed meal | 30 | 50 |
| C | Yeast extract | 10 | 20 |
| D | CaCO_3_ | 5 | 9 |
| E | K_2_HPO_4_ | 0.1 | 0.7 |
| F | FeSO_4_ | 0.02 | 0.2 |
| G | Inoculum amount | 5 | 15 |
| H | Dummy1 | -1 | +1 |
| J | Dummy2 | -1 | +1 |
| K | Dummy3 | -1 | +1 |
| L | Dummy4 | -1 | +1 |

**Table S5** Plackett-Burman experimental design and results

| Run number | A | B | C | D | E | F | G | H | J | K | L | Response  mg/L |
| --- | --- | --- | --- | --- | --- | --- | --- | --- | --- | --- | --- | --- |
| 1 | 60 | 50 | 10 | 9 | 0.7 | 0.2 | 5 | -1 | -1 | 1 | -1 | 214.1 |
| 2 | 40 | 50 | 20 | 5 | 0.7 | 0.2 | 15 | -1 | -1 | -1 | 1 | 167.3 |
| 3 | 60 | 30 | 20 | 9 | 0.1 | 0.2 | 15 | 1 | -1 | -1 | -1 | 207.5 |
| 4 | 40 | 50 | 10 | 9 | 0.7 | 0.02 | 15 | 1 | 1 | -1 | -1 | 123.2 |
| 5 | 40 | 30 | 20 | 5 | 0.7 | 0.2 | 5 | 1 | 1 | 1 | -1 | 115.9 |
| 6 | 40 | 30 | 10 | 9 | 0.1 | 0.2 | 15 | -1 | 1 | 1 | 1 | 107.1 |
| 7 | 60 | 30 | 10 | 5 | 0.7 | 0.02 | 15 | 1 | -1 | 1 | 1 | 208.4 |
| 8 | 60 | 50 | 10 | 5 | 0.1 | 0.2 | 5 | 1 | 1 | -1 | 1 | 227.7 |
| 9 | 60 | 50 | 20 | 5 | 0.1 | 0.02 | 15 | -1 | 1 | 1 | -1 | 248.3 |
| 10 | 40 | 50 | 20 | 9 | 0.1 | 0.02 | 5 | 1 | -1 | 1 | 1 | 152.4 |
| 11 | 60 | 30 | 20 | 9 | 0.7 | 0.02 | 5 | -1 | 1 | -1 | 1 | 218.8 |
| 12 | 40 | 30 | 10 | 5 | 0.1 | 0.02 | 5 | -1 | -1 | -1 | -1 | 96.1 |

**Table S6** The analysis of variance for the Plackett-Burman test

| Source | Sum of Squares | Degree of freedom | Mean square | *F* | *P* | significant |
| --- | --- | --- | --- | --- | --- | --- |
| Model | 30736.67 | 7 | 4390.95 | 34.15 | 0.0021 | ** |
| A | 26320.33 | 1 | 26320.33 | 204.69 | 0.0001 | ** |
| B | 2700.00 | 1 | 2700.00 | 21.00 | 0.0102 | * |
| C | 1452.00 | 1 | 1452.00 | 11.29 | 0.0283 | * |
| D | 133.33 | 1 | 133.33 | 1.04 | 0.3661 |  |
| E | 5.33 | 1 | 5.33 | 0.041 | 0.8486 |  |
| F | 5.33 | 1 | 5.33 | 0.041 | 0.8486 |  |
| G | 120.33 | 1 | 120.33 | 0.94 | 0.3881 |  |
| Residual | 514.33 | 4 | 128.58 |  |  |  |
| Cor Total | 31251.00 | 11 |  |  |  |  |

*F*: Fishers’s function; *P*: Level of significance; *R*^2^ = 0.9835

**Table S7** The design and results of steepest ascent experiment

| Number | Glucose  （g/L） | cottonseed meal（g/L） | yeast extract（g/L） | butenyl-spinosyn（mg/L） |
| --- | --- | --- | --- | --- |
| 1 | 40 | 25 | 5 | 269.6 |
| 2 | 45 | 30 | 10 | 278.1 |
| 3 | 50 | 35 | 15 | 273.5 |
| 4 | 55 | 40 | 20 | 252.6 |
| 5 | 60 | 45 | 25 | 248.9 |

**Table S8** Factors and levels of Central Composite design

| Factors | |  | Experimental Levels | |
| --- | --- | --- | --- | --- |
| number | Factors | -1 | 0 | 1 |
| A | Glucose | 40 | 45 | 50 |
| B | Cottonseed meal | 25 | 30 | 35 |
| C | Yeast extract | 5 | 10 | 15 |

**Table S9** Experimental design and results of CCD

| Number | A | B | C | butenyl-spinosyn（mg/L） |
| --- | --- | --- | --- | --- |
| 1 | -1 | -1 | -1 | 268.7 |
| 2 | 1 | -1 | -1 | 261.5 |
| 3 | -1 | 1 | -1 | 256.2 |
| 4 | 1 | 1 | -1 | 250.7 |
| 5 | -1 | -1 | 1 | 262.8 |
| 6 | 1 | -1 | 1 | 240.9 |
| 7 | -1 | 1 | 1 | 280.2 |
| 8 | 1 | 1 | 1 | 268.8 |
| 9 | -1.682 | 0 | 0 | 275.1 |
| 10 | 1.682 | 0 | 0 | 257.4 |
| 11 | 0 | -1.682 | 0 | 256.2 |
| 12 | 0 | 1.682 | 0 | 264.7 |
| 13 | 0 | 0 | -1.682 | 265.6 |
| 14 | 0 | 0 | 1.682 | 273.8 |
| 15 | 0 | 0 | 0 | 291.3 |
| 16 | 0 | 0 | 0 | 289.8 |
| 17 | 0 | 0 | 0 | 296.9 |
| 18 | 0 | 0 | 0 | 292.1 |
| 19 | 0 | 0 | 0 | 293.3 |

**Table S10** Regression analysis results of the *R* model

| Source | Sum of Squares | Degree of freedom | Mean square | *F* | *P* | Significance |
| --- | --- | --- | --- | --- | --- | --- |
| Model | 4634.91 | 9 | 514.99 | 73.34 | < 0.0001 | ** |
| A | 437.22 | 1 | 437.22 | 62.26 | < 0.0001 | ** |
| B | 97.31 | 1 | 97.31 | 13.86 | 0.0048 | ** |
| C | 59.29 | 1 | 59.29 | 8.44 | 0.0174 | * |
| AB | 15.13 | 1 | 15.13 | 2.15 | 0.1763 |  |
| AC | 55.12 | 1 | 55.12 | 7.85 | 0.0206 | ** |
| BC | 595.13 | 1 | 595.13 | 84.75 | < 0.0001 | ** |
| A^2^ | 1322.80 | 1 | 1322.80 | 188.38 | < 0.0001 | ** |
| B^2^ | 1954.33 | 1 | 1954.33 | 278.32 | < 0.0001 | ** |
| C^2^ | 1053.11 | 1 | 1053.11 | 149.97 | < 0.0001 | ** |
| Residual | 63.20 | 9 | 7.02 |  |  |  |
| Lack of Fit | 36.40 | 5 | 7.28 | 1.09 | 0.4814 | ns |
| Pure Error | 26.80 | 4 | 6.70 |  |  |  |
| Cor Total | 4698.11 | 18 |  |  |  |  |

**Table S11** Correlation analysis results of the experimental model

| Project | Value |
| --- | --- |
| Standard deviation | 2.65 |
| Mean | 270.32 |
| Coefficient of variation | 0.98 % |
| Sum of squared prediction errors | 333.06 |
| model correlation coefficient（*R*^2^） | 0.9865 |
| Adjusted coefficient of determination（*R*^2^_Adj_） | 0.9731 |
| Predictive coefficient of determination（*R*^2^_Pre_） | 0.9291 |
| Relative Accuracy | 25.729 |

**Supplemental References**

(1) Zhao, X.; Hussain, M. H.; Mohsin, A.; Liu, Z.; Xu, Z.; Li, Z.; Guo, W.; Guo, M. Mechanistic insight for improving butenyl-spinosyn production through combined ARTP/UV mutagenesis and ribosome engineering in *Saccharopolyspora pogona*. Front. Bioeng. Biotech*.* 2023, *11*, 1329859.
